# Bottom-up and top-down approaches to understanding oppositional defiant disorder symptoms during early childhood: a mixed method study

**DOI:** 10.1186/s13034-020-00339-1

**Published:** 2020-09-14

**Authors:** Britt-Marie Ljungström, Elisabeth Kenne Sarenmalm, Ulf Axberg

**Affiliations:** 1 Department of Psychology, University of Gothenburg, Box 500, 405 30 Göteborg, Sweden; 2 Institute of Health and Care Sciences, Sahlgrenska Academy at the University of Gothenburg, Box 400, 405 30 Göteborg, Sweden; 3 Research and Development, Skaraborg Hospital, 541 85 Skövde, Sweden; 4 Fakultet for Sosialfag/Faculty of Social Studies, Familieterapi Og Systemisk Praksis/Family Therapy and Systemic Pratice, VID Vitenskapelige høgskole/VID Specialized University, Oslo, Norway; 5 Department of Psychology, University of Gothenburg, Box 500, 405 30 Göteborg, Sweden

**Keywords:** Oppositional defiant disorder, Antisocial behavior, Mixed methods, K-SADS, Bottom–up-top–down

## Abstract

**Background:**

Children with clinical levels of conduct problems are at high risk of developing mental health problems such as persistent antisocial behavior or emotional problems in adolescence. Serious conduct problems in childhood also predict poor functioning across other areas of life in early adulthood such as overweight, heavy drinking, social isolation and not in employment or education. It is important to capture those children who are most at risk, early in their development. The Diagnostic and Statistical Manual of Mental Disorders (DSM–5) is commonly used in clinical settings, to identify children with conduct problems such as oppositional defiant disorder (ODD).This paper presents a cross-sectional study in a clinical setting, and describes behaviors in 3- to 8-year-olds with ODD. Our aim was to investigate whether there were problematic behaviors that were not captured by the diagnosis of ODD, using two different methods: a clinical approach (bottom-up) and the nosology for the diagnosis of ODD (top-down).

**Method:**

Fifty-seven children with clinical levels of ODD participated in the study. The mothers were interviewed with both open questions and with a semi-structured diagnostic interview K-SADS. The data was analyzed using a mixed method, convergent, parallel qualitative/quantitative (QUAL + QUAN) design. For QUAL analysis qualitative content analysis was used, and for QUAN analysis associations between the two data sets, and ages-groups and gender were compared using Chi-square test.

**Results:**

In the top-down approach, the ODD criteria helped to identify and separate commonly occurring oppositional behavior from conduct problems, but in the bottom-up approach, the accepted diagnostic criteria did not capture the entire range of problematic behaviors-especially those behaviors that constitute a risk for antisocial behavior.

**Conclusions:**

The present study shows a gap between the diagnoses of ODD and conduct disorder (CD) in younger children. Antisocial behaviors manifest in preschool and early school years are not always sufficiently alarming to meet the diagnosis of CD, nor are they caught in their entirety by the ODD diagnostic tool. One way to verify suspicion of early antisocial behavior in preschool children would be to specify in the ODD diagnosis if there also is subclinical CD.

## Background

Many children and adolescents in psychiatric care exhibit conduct problems in the form of defiance, aggression and antisocial behavior [1]. Studies have shown that children who demonstrate serious conduct problems in early childhood are at high risk to develop life-long difficulties such as emotional problems and/or antisocial behavior as well as poor functioning across other areas of daily life in early adulthood, such as heavy drinking, overweight, social isolation and not in employment or education [2, 3]. In addition, if the antisocial behavior begins in early childhood and persists through childhood and youth, there is a great risk that it will stabilize and develop into antisocial personality disorder and criminality in adulthood [4]. Therefore, it seems essential to pay attention to childhood-onset of conduct problem and to constantly refine diagnostic instruments to promptly identify those children at highest risk for developing stable disruptive behavior [5, 6]. The present study will focus on conduct problems such as oppositional defiant disorder and conduct disorder in preschool children and early school age.

### Oppositional defiant disorder (ODD), conduct disorder (CD)

In the fifth edition of the Diagnostic and Statistical Manual of Mental Disorders (DSM-5) severe conduct problems are represented primarily by Oppositional Defiant Disorder (ODD) and Conduct Disorder (CD) [7]. ODD is defined mainly by irritable disposition and resistant interactions with authority figures, and is often conceptualized as a disorder of early childhood [8], while CD criteria are essentially designed to describe behavioral problems in older children and adolescents [9]. There are no differences in the DSM diagnostic criteria for girls and boys, but most studies show a higher prevalence of ODD in boys than girls [10], and it has been questioned if the diagnostic criteria for ODD are as clinically useful in girls as in boys [11]. CD is characterized by disregard for social norms and rules, disregard for the rights and well-being of others, and related serious aggression. The DSM-5 defines three subtypes of CD: childhood-onset when at least one symptom of CD occurs in a child under 10 years; adolescent-onset when no symptoms occur prior to age 10; and unspecified onset [7]. In addition callous-unemotional traits (CU-traits) through the Limited Prosocial Emotions (LPE) specifier.

## Different approaches to obtain information

Top-down and bottom-up approaches are two different strategies for processing information [12]. A top-down approach uses an already formulated system or method and breaks the whole into smaller analytical pieces using existing general knowledge to understand the individual case. The DSM, for example, offers a top-down process based on various diagnoses and criteria agreed upon by groups of experts, the DSM committees [13]. In the text above, we have briefly described how children´s and adolescent´s problems are classified according to diagnostic categories, where each diagnostic category implies a taxonomic construct. To determine whether the criteria for the DSM categories are met, each symptom in the different diagnosis are judged absent or present. A top-down approach is necessary to interpret and understand what we perceive and is often used in research. However, one criticism of the DSM is that its categories and criteria are predetermined [14]. The top-down orientation makes it difficult to discover the unexpected by not allowing the formation of new knowledge. A bottom-up approach uses individual cases to create new general knowledge; it progresses from individual elements to build a view of the whole, piecing data together until a larger picture is formed [13]. Thus, a bottom-up approach is also important since it can help us to refine and improve our knowledge systems. This type of information is of course more subjective and random, but it can be tested for validity and utility as markers for important characteristics, for robustness across samples and for developmental course [13, 15]. Wakschlag and colleagues [9], argued the importance of integrating developmental psychology and studies based on clinical research for generating and testing developmentally specified nosology. Their work is a bottom-up way of understanding behavioral problems of young children. As a framework for a clinical and developmental understanding of disruptive behavior in preschool-age children, they proposed a multi-dimensional model to define the core dimensions of disruptive behavior problems (DBP) [9, 16]. The four core dimensions they created for disruptive behavior were Temper Loss (clinical indicator: frequent and intense temper tantrums), Aggression (frequent, hostile, and proactive aggression), Noncompliance (stubborn and pervasive noncompliance), and Low Concern for Others (enjoying distressing and provoking others) [16].

Oppositional defiant disorder (ODD) early in a child’s life should be considered an important marker of a wider pattern of dysfunctional regulation of behavior and emotions that can predict the development of severe psychopathology [8, 17]. Many studies conducted on ODD in children are top-down studies based on defined diagnostic criteria [6, 18]. However, using only a top-down perspective might impose limitations in the information we obtain. To broaden the survey of problematic behaviors in preschool and early school-aged children, our aim in the present study, is to go more in-depth and beyond the diagnostic criteria by combining a bottom-up and a top-down approach in a mixed-method study, with a qualitative/quantitative (QUAL + QUAN) design. We will investigate whether there were problematic behaviors in children aged 3–5 and 6–8 years that were not captured by the diagnosis of ODD, using two different methods: a clinical approach (bottom-up) and the nosology for the diagnosis of ODD (top-down).

## Aims

The overall purpose of this study was to explore mothers’ descriptions of their children (bottom-up) and compare them with descriptions that emerged through a standardized semi-structured diagnostic interview (top down). Specifically, we aimed to answer the following questions:

1. How do mothers describe their children when they are asked to identify the major problems they are experiencing with their child (bottom-up)? How do mothers describe their children when they answer a standardized semi-structured diagnostic interview (top-down)?
2. Are there any differences between gender and ages in the mothers’ descriptions in the bottom-up and the top-down approach?
3. What kinds of convergences, divergences, contradictions and associations in results are found between the bottom-up and top-down approaches based on the mixed method in this study? Does it add any additional dimension using mixed methods in research on ODD?

## Methods

### Procedure

This study was part of the first RCT in Sweden to evaluate the effectiveness of the American parent training program, *The Incredible Years* [19]. The study was approved by Gothenburg University and The Sahlgrenska Academy Ethics committee (D:nr Ö 669-03). Informed consent was obtained from all.

Parents of children with disruptive behavior signed up for parent training groups, which were provided at two Child and Adolescent Mental Health Services in Skaraborg. A semi-structured interview, The Kiddie-SADS-Present Version (K-SADS) [20], was completed with the mothers to assess whether the children met the criteria for ODD and other psychiatric diagnoses. From the very beginning, the ambition was to interview both parents, but very often only mothers appeared. Therefore, following the usual procedure of the child and adolescent mental health services, were the study was carried out, and to obtain a more homogeneous sample, a decision was made early in the research process to interview only mothers. These interviews were conducted by three experienced clinical psychologists, trained in the K-SADS interview. Each interview took approximately 3 h. The interviews were recorded and coded.

### Participants

To participate in the study, children had to be 3 to 8 years old and meet the DSM-IV criteria for ODD through the diagnostic interview K-SADS. Parents had to understand Swedish well enough to fill out the forms used in the study and give their permission for their child to participate in the study. Parents were invited to participate in *The Incredible Years* training if their children’s problems met that program’s inclusion criteria. Parents of 4 children declined to participate after an initial telephone interview. Altogether, 62 children and their parents were included in the study. Parents of 5 children in the study dropped out. Parents of 57 children (11 girls, 19%; 46 boys, 81%; 1:4) agreed to continue in the study. The ages of the children were 3–5 years, 35%; 6–8 years, 65%. Of the children in the sample, 61% lived with their biological mother and father (or adoptive parents). Both of the parents of 91% of the children had been born in Sweden. The educational level of mothers in the sample was in line with the level of education of mothers in the general population. Most of the mothers, 77%, were employed or in studies. According to Hollingshead´s four factor index of social position [21], 41% were classified as low socioeconomic standard. Co-morbidity was common, 54% of the children with ODD, also met the criteria for attention deficit hyperactivity disorder (ADHD), one child met the criteria for CD and 14% an anxiety disorder. Other diagnoses were tics, Tourette`s syndrome, maladaptive stress, enuresis, and encopresis. Some of the children also had autistic traits. Twenty-one percent fulfilled criteria for three or more diagnosis. Sixty-four percent of the girls and 28% of the boys had ODD only (*p* = 0.038).

### Measures and interviews

#### QUAL interviews with open-ended questions

Initially, the mothers were asked open-ended questions to describe the major problems they experienced with their children. These questions were asked before the structured K-SADS interview, i.e. before anything about diagnosis or diagnostic criteria was mentioned and formed the basis of the qualitative analysis in the present study.

#### QUANT component with K-SADS

K-SADS is a semi-structured diagnostic interview with three different parts: an initial background interview, a diagnostic screening interview, and a section in-depth questions about the different diagnoses [20]. The K-SADS consists of several questions aimed to capture the essence of each criterion in the different DSM-IV diagnoses. The interviewer assessed the parents’ answers about the severity of the problem on a 4-point scale, on which 0 = not enough information, 1 = the problem does not exist, 2 = below the threshold and 3 = above the threshold (if the problematic behavior occurred at least 4–5 times per week). If any diagnostic criterion was met in the screening interview, supplemental questions were asked to decide whether the child met the full criteria for the specific diagnosis. Because the present study focused on conduct problems, all the supplemental questions about existing ADHD, ODD, and CD were asked. The interview is designed for children aged 6 to 17, but the questions for ADHD and CD were adapted by the psychologists in the criteria it required, for children aged 3 to 5 years (35% of the sample). The questions about ODD symptoms were unchanged because they were judged well adapted to children aged 3 to 5 years.

### Data analyses

**Fig. 1:**
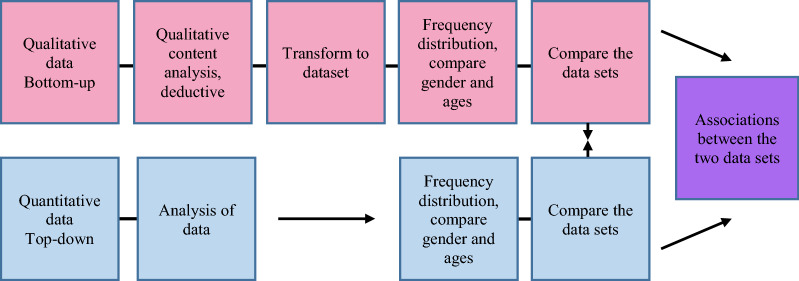
Convergent parallel design; QUAL + QUAN

The present study is an exploratory study using mixed methods with a convergent parallel design QUAL + QUAN, in which qualitative and quantitative data were collected concurrently [22]. Figure 1 displays the mixed method convergent parallel design schematically.

#### QUAL analyses

The qualitative content analysis was performed, following the steps suggested by Graneheim and Lundman [23] and Elo and Kyngäs [24]: Mothers (n = 57) described their children’s major problems, and the interviewer wrote down these descriptions. The transcripts of the formulated problems were read through several times to learn “what was going on” and to discern patterns. Each separate problem formed a meaning unit for the analysis. The transcript of the children’s problems was then condensed. The next step was to code these condensed meaning units. The codes were then grouped into subcategories, and the subcategories were grouped into larger categories. The analysis focused on the manifest content and the codes; subcategories and categories were limited to textual content and did not involve interpretation of the underlying meaning of the text [23]. One of the co-authors was an expert on DBDs and the other co-author was an expert in the qualitative method. The process of developing codes and creating subcategories and categories was laborious as the material was passes back and forth between the three authors, followed by discussions until consensus regarding the codes, subcategories, and categories was reached. Finally, based on the categories, three different themes became clear.

#### QUAN analysis

To test interrater reliability among the three psychologist who conducted the K-SADS interviews, one third of the interviews concerning the diagnostic parts were coded by two coders; intraclass correlation coefficient (ICC) was calculated for the ODD total score and the total symptom score (rater 1 and 2, and rater 1 and 3; two-way mixed; absolute agreement; average measure). Interrater reliability was excellent at over 0.95 for all combinations.

We made a description of frequencies from the data set of the eight criteria for ODD. Girls and boys, and ages-groups were compared on the distribution of the eight criteria using Chi-square test. We also compared boys and girls, and age groups in the qualitative content analysis using Chi-square.

#### Mixed-method analysis

The third step in the mixed method convergent parallel design was to merge the two data sets together (QUAL + QUAN) [22], to achieve a more complete understanding of ODD. Even if each part of QUAL and QUAN would be complete in themselves, we were interested in finding and analyzing the interface between the two [25]. This process was carried out in two different ways. First, we put the data sets next to each other and compared for contradictions, divergences, and convergences. In this way, we achieved a triangulation of the results of the bottom-up and top-down analyses [26]. Next, we looked for associations between the two data sets using Chi-square test. One of the ideas of the study was to explore possible associations between the data sets, therefore we chose not to correct for multiple comparisons. To allow individual comparisons between mothers’ descriptions; their most serious problems with their children (bottom-up) and their responses to the diagnostic questions (top-down), we registered each child with the themes and categories that their problems belonged.

Because the analyzed data were categorical or ordinal, we calculated the frequencies and proportions and used non-parametric statistical tests for comparisons between the groups, Mann–Whitney test for ordinal data, and Chi-square test, or Fisher test for categorical data, when cells were smaller than 5.

## Results

### QUAL results

**Fig. 2:**
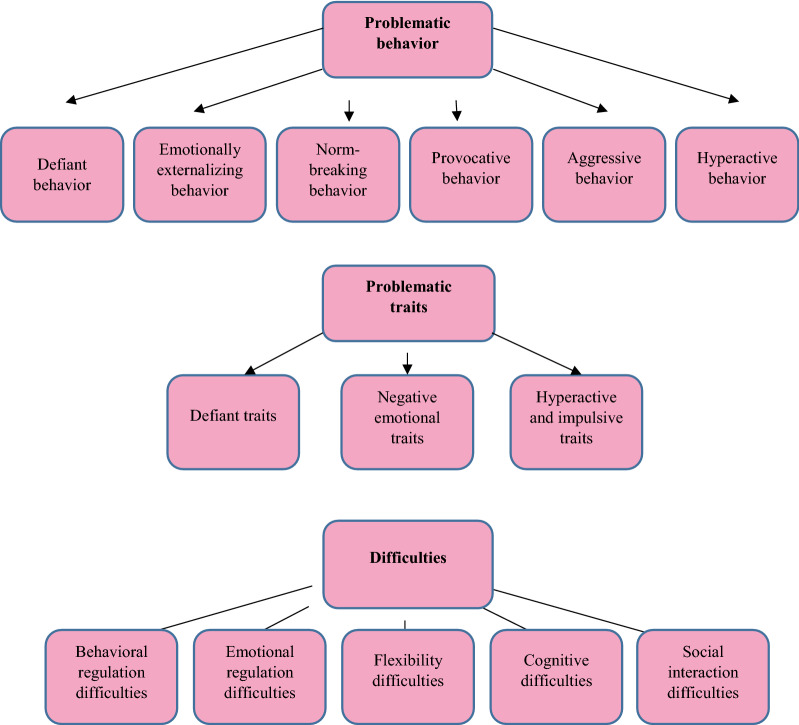
Results from the qualitative content analysis showing three different themes; Problematic behavior, Problematic traits, and Difficulties and the categories within each

Of 225 problems formulated from mothers’ descriptions, we used 220 in the qualitative content analysis. The five problems discarded were judged to differ from research question. Because the qualitative analysis was limited to textual content, we have been responsive to in which way mothers verbally described their children. The more familiar the material became, the clearer it was that the mothers described three main things, which formed the three different themes in the study. Mothers described Problematic behavior—what the children do (e.g., *constantly fighting, attacking those who pass*), the Problematic traits of the children—how the children are (e.g., *is frustrated and angry*), and the child’s Difficulties—what they could not do (e.g., *has difficulty mastering his temper*). Sometimes these themes seemed to interweave as above; *is frustrated and angry* and *has difficulty mastering his temper,* but in the analysis we separate them as mothers´ descriptions of how they described their child more in terms of traits (*is frustrated and angry*) and how they saw their child´s difficulties (*has difficulty mastering his temper*). Of these, three main themes were *Problematic behavior* (44%), *Problematic traits* (25%), and *Difficulties* (31%.) Fig. 2 shows how the problems mothers described were analyzed and sorted into themes, each with various categories.

#### Problematic behaviors

**Fig. 3:**
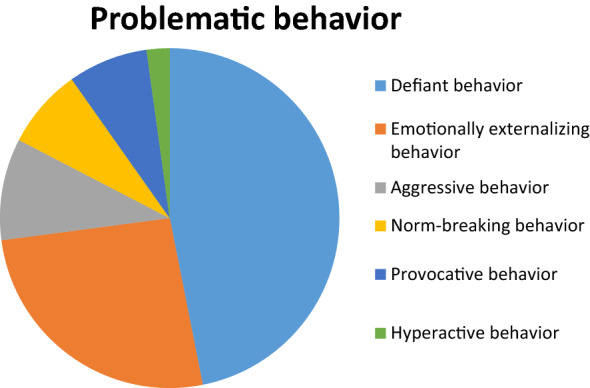
Description of problematic behaviors

The largest group of reported problems were *Problematic behaviors* that mothers experienced as very stressful. During the analysis, six categories of problematic behavior emerged: *defiant, emotionally externalizing, norm-breaking, provocative, aggressive,* and *hyperactive behaviors.* The problems that most mothers experienced as very difficult were *defiant behaviors* (see Fig. 3). Only a few mothers described *hyperactive behavior* as a major problem, even though 54% of the children met the criteria for ADHD in K-SADS interview.

We found three different qualities of *Defiant behavior* in the analysis; disobedience, inflexibility and rebelliousness. The mothers described *Emotionally externalizing behavior* as aggressive outbursts, severe outbursts of anger, temper tantrums, and screaming and shouting. *Norm-breaking behavior* was divided in two subcategories: dishonesty (lying, fibbing, and blaming others) and negative behavior (breaking things, running away from home, and drawing on walls). *Aggressive behavior* was sorted into three subcategories according to whether children violated siblings, parents, or peers. *Provocative behavior* included teasing, but more commonly conflict-seeking, conflict-seeking and -initiating, fighting with siblings, and fighting with peers. The mothers described *Hyperactive behaviors* as over-activity and restlessness. These categories are described in Table 1.

**Table 1:** Problematic behavior (PB) and Problematic traits (PT) are two of the three themes from the qualitative content analysis. The figure presents the different categories with their subcategories and some examples of different problems, described by the mothers

Category (PB)	Subcategory/dimensions	Examples of problems
Defiant behavior	Disobediance	Does not obey; Does not obey when choosing clothes; Causes trouble when eating; Does not do homework; Creates conflicts in everyday routine situations; Does not listen; Does not mind me
	Inflexibility	Has his own rules; Wants to decide everything himself; Wants to do things in his own way; Has strange ideas; Has fixed ideas
	Rebelliousness	Powerstruggles with mother daily; Constant contrary to parents; Test limits; Protesters; Refuses to follow rules; Refuses to follow corrections; Refuses to stop; Refuses to cooperate; Constant struggles each time when doing things
Emotionally externalizing behavior	Outbursts	Aggressive outbursts; Severe outbursts of anger; Temper tantrums
	Noise	Screaming and shouting
Norm-breaking behavior	Dishonesty	Lying; Fibbing; Blaming others
	Negativ behavior	Running away from home; Breaking things; Drawing on walls
Provocative behavior	Teasing	Teases little brother; Treats brother bad
	Conflict seeking	Seeking and initiating conflict; Fighting with siblings; Fighting with peers
Aggressive behavior	Violates siblings	Hits little brother; Attacks younger sister; Physical fights with siblings
	Violates parents	Attacks mother; Wants to hurt dad
	Violates peers	Attacks others, Constantly fighting; Attacks those who pass
Hyperactive behavior	High activity	Overactivity; Anxiety with restlessness
***Category (PT)***		
Defiant traits	Disobediant traits	Is obstinate; Bossy; Insistent; Loudmouthed; Contentious
	Inflexible traits	Is stubbhorn; Headstrong; Strong-willed
	Rebellious traits	Is impertinent; Disrespectful
	Provocative traits	Is provocative; Disorderly
Negative emotional traits	Internalized traits	Is tense and anxious; Afraid; Constantly Worried; Has fears; Is afraid to fail; Is sad; Does not feel well; Has low self-esteem
	Negativistic traits	Is unpleasant; Jealous; Negative; Is grumpy; Frustrated; Never satisfied; Whiny
	Aggressive traits	Is angry; Aggressive; Frustrated and angry; Aggressive with no provocation
Hyperactive and impulsive traits	High activity traits	Is restless; Active; Intense; Extremely impatient
	Impulsive traits	Is intrusive; Impulsive

#### Problematic traits

Many of the problem’s mothers formulated expressed three main categories of *Problematic traits* within the child: *defiant, negative emotional,* and *hyperactive/impulsive traits.* More mothers thought that defiant traits were a bigger problem than negative emotionality and hyperactivity/impulsivity. The lower part of Table 1 shows the qualitative content analysis of problematic traits in children.

#### Difficulties

Of all the problems the mothers formulated, nearly one third described *Difficulties* within the child. We sorted these difficulties into five different categories: *behavioral regulation, emotional regulation, flexibility, cognitive* (the largest category)*,* and *social interaction difficulties*. The five categories with their subcategories and examples of problems are shown in Table 2.

**Table 2:** Five different categories of Difficulties (D) with subcategories and problems, as described by the mothers in the study

Category (D)	Subcategory/dimension	Examples of problems
Difficulties with behavioral regulation	Difficulties with hyperactivity	Cannot sit still; Does not know when to stop
	Trouble with compliance	Has difficulty being corrected; Has difficult to accept no; Gets upset being told off
	Difficulties with independence	Cannot play alone and self-entertain; Requires constant attention; Always need to be in centre
Difficulties with emotional regulation	Difficulties regulating anxiety	Is concerned about mother; Wants to be very near parents when going to sleep; Difficult to separate when going to school
	Difficulties regulating mood	Has difficulty mastering temper; Easily angry; Loses control over temper
Difficulties with flexibility	Difficulties with changes	Difficulties with different transitions; Needs much preparation; Sensitive to change; Unsure in new situations
	Becomes fixated	Hard to break off activities; Rigid in many situations
Cognitive difficulties	Lack of concentration	Cannot concentrate; Is careless with everything
	Inattention	Has difficulty listening; Is difficult to reach and talk to
	Difficulties with intellectual capacity	Does not always understand; Does not understand consequences
Difficulties with social Interaction	Limited social skills	Can only interact with one person at a time; Has difficulty with social skills
	Limited in acting in groups	Has difficulty acting in a group; Has difficulty interacting with peers; Has difficulty with social interactions in school

### QUAN results

A descriptive compilation was made based on the diagnostic questions in the K-SADS. Table 3 shows the distribution of the various ODD criteria. We also compared boys, girls and ages in terms of the distribution of diagnostic criteria (Table 3) and the themes and categories in the qualitative content analysis (Table 4).

**Table 3:** The distribution of the different ODD criteria. Comparisons between boys and girls and between age-groups

Criteria, n (%)	Study group, n = 57	Girls, n = 11	Boys, n = 46	p-value^a^	3–5 years, n = 21	6–8 years, n = 36	p-value^b^
Often loses temper							
No (1–2)	7 (12.3)	2 (18.2)	5 (10.9)	0.507	0	7 (19.4)	0.039*
Yes (3)	50 (87.7)	9 (81.8)	41 (89.1)		21 (100)	29 (80.6)	
Often argues with authority figures							
No (1–2)	7 (12.3)	1 (9.1)	6 (13.0)	0.720	2 (9.5)	5 (13.9)	0.628
Yes (3)	50 (87.7)	10 (90.9)	40 (87.0)		19 (90.5)	31 (86.1)	
Often actively defies or refuses to comply.							
No (1–2)	13 (22.8)	5 (45.5)	8 (17.4)	0.046*	6 (28.6)	7 (19.4)	0.428
Yes (3)	44 (77.2)	6 (54.5)	38 (82.6)		15 (71.4)	29 (80.6)	
Often deliberately annoys others							
No (1–2)	23 (40.4)	1 (9.1)	22 (47.8)		8 (38.1)	15 (41.7)	
Yes (3)	34 (59.6)	10 (90.9)	24 (52.2)	0.037*	13 (61.9)	21 (58.3)	0.791
Often blames others for his or her mistakes.							
No (1–2)	23 (40.4)	4 (36.4)	19 (41.3)		6 (28.6)	17 (47.2)	
Yes (3)	34 (59.6)	7 (63.6)	27 (58.7)	0.764	15 (71.4)	19 (52.8)	0.166
Is often touchy or easily annoyed							
No (1–2)	15 (26.3)	4 (36.4)	11 (23.9)	0.455	9 (42.9)	6 (16.7)	0.030*
Yes (3)	42 (73.7)	7 (63.6)	35 (76.1)		12 (57.1)	30 (83.3)	
Is often angry and resentful							
No (1–2)	19 (33.3)	4 (36.4)	15 (32.6)	0.812	9 (42.9)	10 (27.8)	0.244
Yes (3)	38 (66.7)	7 (63.6)	31 (67.4)		12 (57.1)	26 (72.2)	
Is often spiteful or vindictive							
No (1–2)	40 (70.2)	7 (63.6)	33 (71.7)	0.717	17 (81.0)	23 (63.9)	0.235
Yes (3)	17 (29.8)	4 (36.4)	13 (28.3)		4 (19.0)	13 (36.1)	

1 = No problem, 2 = below threshold, and 3 = above threshold

^a^ p-value considers comparisons between boys and girls

^b^ p-value considers comparisons between the two age groups

* Statistically significant according to Chi-2 test (Fisher’s test when n < 5 in some of the cells)

**Table 4:** Results from the qualitative content analysis, with themes and categories. The table also shows comparisons between boys and girls and between the two age-groups

Theme and Category, n (%)	Girls, n = 11	Boys, n = 46	p-value^a^	3–5 years, n = 21	6–8 years, n = 36	p-value^b^
Problematic behaviors	11 (100)	32 (69.6)	0.049*	15 (71.4)	28 (77.8)	0.591
Problematic traits	8 (72.7)	24 (52.2)	0.315	10 (47.6)	22 (61.1)	0.322
Difficulties	5 (45.5)	34 (73.9)	0.068	13 (61.9)	26 (72.2)	0.419
Norm-breaking behavior	2 (18.2)	2 (4.3)	0.095	1 (4.8)	3 (8.3)	0.878
Provocative behavior	0 (0)	6 (13.6)	0.334	2 (9.5)	4 (11.1)	0.823
Aggressive behavior	2 (18.2)	4 (8.7)	0.586	2 (9.5)	4 (11.1)	0.823
Defiant behavior	10 (90.9)	23 (50.0)	0.017*	11 (52.4)	22 (61.1)	0.440
Emotionally externalizing behavior	6 (54.5)	16 (34.8)	0.248	7 (33.3)	15 (41.7)	0.480
Hyperactive behavior	1 (9.1)	1 (2.2)	0.357	1 (4.8)	1 (2.8)	0.710
Negative emotional traits	3 (27.3)	18 (39.1)	0.728	5 (23.8)	16 (44.4)	0.150
Defiant traits	6 (54.5)	7 (15.2)	0.006*	5 (23.8)	8 (22.2)	0.935
Hyperactive traits	0 (0)	6 (13.0)	0.334	2 (9.5)	4 (11.1)	0.823
Social difficulties	1 (9.1)	10 (21.7)	0.434	2 (9.5)	9 (25.0)	0.179
Cognitive difficulties	1 (9.1)	16 (34.8)	0.144	7 (33.3)	10 (27.8)	0.708
Inflexibility difficulties	1 (9.1)	8 (17.4)	0.671	4 (19.0)	5 (13.9)	0.715
Emotional regulation difficulties	1 (9.1)	6 (13.0)	0.703	3 (14.3)	4 (11.1)	0.754
Behavioral regulation difficulties	1 (9.1)	10 (27.1)	0.434	4 (19.0)	7 (19.4)	0.931

^a^p-value considers comparisons between boys and girls

^b^p-value considers comparisons between the two age groups

* Statistically significant according to Chi-2 test (Fisher’s test when n < 5 in some of the cells)

There were significant differences in criterion 3, *Often actively defies or refuses*; boys defied or refused significantly more often than girls (*p* = 0.046), and in criterion 4, *Often deliberately annoys others*, with girls deliberately annoying others significantly more often than boys (*p* = 0.037). There were significant differences between age groups; children aged 3–5 years lost their tempers more often but were less often touchy or easily annoyed than the other age group (*p* = 0.039 vs. *p* = 0.030).

In the distribution of the themes and categories there were some differences between boys and girls. In the themes, girls were significantly more often described to have *Problematic behaviors* (*p* = 0.049). In the categories, girls were significantly more often described to have *defiant traits* (*p* = 0.006) and *defiant behavior* (*p* = 0.017). When comparing the different age groups, we found no significant differences between ages 3–5 and ages 6–8.

### Mixed methods result

#### Associations between the two data sets

There was an association between the two variables *Often angry and resentful* (DSM criteria) and the category of *norm-breaking behavior* (*p* = 0.029) in which children with norm-breaking behavior seemed to be less angry and resentful (0% vs. 72%).

There was also an association between *Often argues with authority figures* and the category of *difficulties with cognitive skills* (*p* = 0.022) in which children with cognitive difficulties seem to argue less with authority Figs. (71% vs. 95%).

A tendency for association was found between the criterion *Often actively defies or refuses to comply with adults’ requests or rules* and the category of *aggressive behavior* (*p* = 0.100). Children who showed aggressive behavior toward others seemed less often to actively defy or refuse to comply with adult’s requests or rules (50% vs. 80%).

We also expected that children who fulfilled the criteria *Often spiteful and vindictive* or *Often angry and resentful* should be associated with the category of *aggressive behavior*, but no such association was found (*p* = 0.219; *p* = 0.390).

## Discussion

The overall purpose of this study was to explore mothers’ descriptions of their children (bottom-up) and compare them with the descriptions that emerged through a standardized diagnostic interview (top-down). To make this comparison possible, we used a mixed method design. When comparing problem descriptions and diagnoses between bottom-up and top-down approaches, we found both convergent and divergent results.

### Convergence between bottom-up and top-down

For the ODD diagnosis as a whole, bottom-up and top-down converged. Two of the themes from the qualitative content analysis, *Problematic behavior* and *Problematic traits*, correlated well with the DSM’s construction of the ODD diagnosis in terms of behaviors (e.g., often loses temper, often argues, often blames others) and descriptions of traits (e.g., is often touchy, spiteful, angry, and resentful). The third theme, *Difficulties*, described regulatory difficulties many of these children had, showing comorbidity with other DSM diagnoses such as ADHD, depression, anxiety, and autistic traits, which converged with the diagnostic interviews. There was also convergence between top-down and bottom-up descriptions of the essential qualities of ODD, expressing both behavioral and emotional disturbances.

Three of the criteria that seem to be core symptoms in the ODD diagnosis overlapped very well with the categories and subcategories in the bottom-up analysis. The criterion *Often actively defies or refuses to comply with adults’ requests or rules* equated with the category *defiant behavior*, which was the most common problem mothers reported, describing children who create conflicts in everyday routine situations and who refuse to follow corrective instruction. *Often loses temper* was also one of the two most common criteria in DSM met by children with ODD, with good agreement with *emotionally externalizing behaviors.* This category was the second largest group of problems mothers mentioned, describing tantrums, outbursts, and screaming and shouting, which matched the criterion well. The third criterion with good agreement between top-down and bottom-up was *Often angry and resentful*. Mothers reported as a major problem that their children had aggressive traits and described them as angry, frustrated and angry, aggressive, and prone to unprovoked aggression, but no mother described their child as resentful, which is included in the diagnostic description. Both approaches, however, highlighted a group of children with ODD who have an ongoing angry mood.

There was also convergence between top-down and bottom-up approaches in the category of *Difficulties*. A large group of children with ODD are immature in their cognitive, social, and emotional development [27, 28]. When mothers in the present study shared their experiences of the largest problems, 68% highlighted the various constraints their children faced (74% of the boys, 46% of the girls). They described deficits in emotional regulation, attention regulation, and behavioral control. Some children also had limitations in intellectual capacity and/or difficulty with flexibility. Assessment on the K-SADS interview revealed that 54% met the criteria for ADHD or ADHD UNS, showing deficits in attention and behavioral regulation. When children with regulation deficits and neurological immaturity are exposed to requirements they are not yet mature enough to cope with, their defiant behavior often occurs at the intersection of demands to self-regulate and their inability to do so [29]. Furthermore, the inflexibility in the bottom-up category of defiant traits and the children´s aggressive behaviors could also be early signs of autism. It is important not to consider the children as simply brutal and defiant; instead we should be aware that many may have developmental neurological difficulties and to a large extent depend upon adaptions and support from their environment.

### Divergences between bottom-up and top-down

In contrast to the DSM, which employs a categorical system, the bottom-up process allows more multi-dimensional thinking, as seen in the case of the category *defiant behavior*. Mothers in the study described three different qualities or dimensions of defiance in their children. The first dimension, *disobedience*, included children’s attempts to ignore and/or act against their parents’ directives. This typically defiant behavior occurs when children disobey, do not listen, and generate conflicts during everyday routine situations. The second defiant dimension was *inflexibility*. These children had difficulty adapting to the demands of their surroundings. They had their own rules, wanted to do things their own way, and had strange and fixed ideas. They expected to live on their own terms and became very frustrated when things did not go their way. They seemed to be more rigid in their defiant behavior. The third dimension of defiance was *rebelliousness*. This dimension seems to be the most severe of the three. The rebellious dimension includes more active refusals and resistance against parents. Mothers described power struggles, refusals, and behaviors associated with a strong negative affect and more aggression in refusing to comply with adults’ requests or rules, which is in the line with Wakschlag et al. [9]. Our findings show notable variations in the quality, severity, and intrinsic degrees of aggressiveness and inflexibility in all three dimensions of defiant behavior. In children with defiant behavior, further investigations are warranted to access the quality of the behavior, which could contribute to better adapted interventions for children with more serious defiant behaviors.

Second, in the diagnostic interview, 74% of mothers responded affirmatively to the item *Is often touchy or easily annoyed* as a problem on the clinical level. In the bottom-up description, however, no mother said spontaneously that her child was touchy or easily annoyed. Instead, they described their children as grudging (unpleasant, jealous, negative, grumpy) and displeased (frustrated, never satisfied, whiny). We called this category of answers negativistic traits. This category seemed to express emotions of dissatisfaction (displeased and grudging) rather than a touchy mood.

There was also a divergence between approaches in the results for antisocial behavior. The mothers reported antisocial behavior in the bottom-up approach, but the ODD criteria did not catch these behaviors. Aggressive behavior (physical violation), together with provocative behavior (seeking and initiating physical conflict), and/or norm-breaking (destroying things, running away, lying), were presented as major problems in about one quarter of the children in this study. Mothers described children who had physically attacked parents, peers, and siblings. Several studies have attempted to identify the characteristics of children with ODD who develop CD and antisocial personality [30–32]. Subthreshold CD symptoms have been identified as predictive [8], and persistent physical fighting is particularly important [33, 34].

Clinical psychologists sometimes seem to lack support in identifying such children. However, it might be inappropriate to use the criteria for a diagnosis of CD in young children. Approximately a quarter of CD symptoms seem to be developmentally impossible in early childhood and approximately one third are developmentally improbable in preschoolers [9]. In the present study, only one of 57 (< 2%) children with ODD also met the full criteria for CD, and this child was in the oldest age group of 8 years. The present study has shown that aggressive, norm-breaking, and provocative behaviors manifest in preschool and early school years are not caught in their entirety by the ODD diagnostic tool, nor are they always sufficiently alarming in early childhood to meet the diagnosis of CD (three criteria need to be met for a diagnosis). Furthermore, some of the children also displayed traits, similar to CU-traits and impulsive traits, which might increase the risk to develop conduct problems and later an antisocial personality [35]. DSM diagnostic tools seem sometimes to be too blunt and not adapted to capture emerging symptoms of severe conduct problems in very young children; this study shows a gap between ODD and CD criteria and diagnoses for the youngest children. In the top-down approach the ODD criteria were found to help identify and separate commonly occurring oppositional behavior from conduct problems, but in the bottom-up approach the accepted criteria did not capture the entire range of problematic behaviors, especially aggressive, provocative, and norm-breaking behaviors that risk developing into persistent antisocial behavior. One way to verify suspicion of early antisocial behavior in preschool children would be to have the possibility to specify in the ODD diagnosis, if there is also subclinical CD (one or two criteria), in the same way as in the CD diagnosis where it is possible to retrospectively specify whether the individual has shown at least one CD symptom before the age of ten (child-hood onset type).

Comparing the content of reported problem behaviors (bottom-up), we found similarity between our descriptions of *Problematic behaviors* and the dimensions described by Wakschlag and collegues [16]. Wakschlag’s four core dimensions for disruptive behavior: Noncompliance, Temper Loss, Aggression, and Low Concern for Others correspond well with our four categories of *defiant*, *emotionally externalizing*, *aggressive*, and *provocative* behaviors*.* However, in addition to the dimensions proposed by Wakschlag and colleagues [16], and the criteria for ODD, we also found a group with norm-breaking behavior.

### Associations

An important part of the mixed-method analysis is to merge the two data sets [22]. Although the sample size was small, there appeared to be a distinction between overt and covert externalizing behavior in this study. These results are similar to those of Loeber and Burke [32], who described three different pathways into antisocial behavior: 1. Overt Pathway, beginning with annoying others and bullying, moving on to more aggressive behaviors such as physical fighting, gang fighting, and rape; 2. Covert Pathway, beginning with lying and shoplifting, leading to vandalism, pick-pocketing, and serious delinquency (theft, burglary); and 3. Authority Conflict Pathway, beginning with stubborn behavior, then disobedience, staying out late, running away, and finally truancy and avoiding authority.

In the present study, several of the behaviors described above, such as annoying others, fighting, lying, destroying things, disobedience, running away and avoiding authority, were represented among the children and were clearly visible in the qualitative content analysis. Furthermore, children with norm-breaking behavior seemed to be less angry and resentful, while children who showed aggressive behavior toward others seemed less often to actively defy or refuse to comply with adults’ requests or rules. The data are based on small groups and these findings merit further research. However, they suggest that maybe it could be possible to trace different pathways of externalizing behavior even in children as young as 3–8 years.

### Differences between girls and boys

While, relatively few studies have investigated differences between boys and girls when it comes to ODD criteria, one of our aims in this study was to examine whether there seemed to be any differences between gender and between bottom-up and top-down approaches. We found no considerable difference between boys and girls in the different ODD criteria or the 14 categories from the content analysis. Thus, ODD seems to be expressed quite similarly in boys and girls. However, some small differences were found that is needed to be mentioned.

In the top-down approach, boys actively defied or refused to comply with adult’s requests or rules more often than girls. In the bottom-up analysis, it was exactly the opposite. Defiant behavior and defiant traits were more often reported as major problems among girls than boys. These contradictory results might be explained by greater social acceptance of defiance in boys than in girls, even when boys are more often and more strongly defiant. If defiant behavior and traits in girls are less socially accepted and considered more problematic, disobedience in girls is probably more noticeable and more likely to be reported by mothers as a major problem, even though these behaviors are more frequent among boys. These results, however, do not fully correspond with Wright and colleagues, who found that only fathers (not mothers) had less tolerance for daughters’ than for sons’ DBD behaviors [36]. However, due to the small sample seize in the present study the results should be interpreted cautiously.

Another difference between boys and girls was found for the criterion *Often deliberately annoys others.* The mothers´ bottom-up descriptions didn´t tally with their answers on the diagnostic question about annoying others. In the diagnostic interview, girls deliberately annoyed others significantly more often than boys. Studies have shown that girls are significantly more relationally aggressive than boys, and this sex difference is apparent as early as the preschool years [27]. Girls tend to engage in higher levels of both proactive and reactive relational aggression compered to physical actions [37]. This indicates that even in early childhood, relational aggression appears to be the modal type of aggression for girls. Comparing results from the bottom-up and top-down analyses, there seem to be several levels of *annoying* severity; teasing and annoying others are on the border of more normative behavior levels, while seeking and initiating conflict (for boys) and relational aggression (for girls) might be on a more clinical level. The differences between boys’ and girls’ disruptive behaviors might be most visible on this criterion. The different provocative behaviors, boys’ and girls’ distinct expressions need to be further explored and perhaps more clearly defined based on diagnostic criteria.

### Differences between ages

In the bottom-up description, we found no significant differences in symptoms between ages 3–5 and 6–8. However, using the top-down approach 3- to 5-year-olds were more likely to *often lose temper*, and 6- to 8-year-olds were *touchy or easily annoyed* significantly more often than children 3- to 5-year-olds. Interestingly, negative emotional traits were described in the bottom-up approach as grudging (unpleasant, jealous, negative, grumpy) or displeased (frustrated, never satisfied, whiny), while no mother spontaneously described their children as touchy, easily annoyed or resentful. This might indicate that the diagnostic criteria that describe irritability (*touchy or easily annoyed* and *angry and resentful*) are more appropriate to describe older children, while grudging and displeased are a quality of descriptions more appropriate for younger children. This might be something to explore further in future studies.

There is also frequency criterion of the diagnosis in the DSM-5, providing guidance on minimum symptom frequencies for different age groups. For children younger than 5 years, the behaviors should occur on most days for a period of at least 6 months, and for those aged 5 years or older, the behavior should occur at least once peer week [7]. Our results from top-down and bottom-up approaches raise questions about the frequency criteria in DSM-5. Are there a risk that we over-diagnose children from 5 years with ODD? According to Wakschlag and colleagues, temper tantrums seem to be more common in preschool-aged children [38]. Our findings is in line with this. The frequency of other symptoms appears to be more constant between the age groups in the present study. It may be, that differences in frequency of disruptive behavior symptoms between younger and older children are greater in community samples than in clinical samples, where the rates of externalizing behavior continue to be high for this group of children.

### Mixed methods design

An important purpose with mixed methods is to investigate convergences and divergences in the interface of the results of the two methods. Through the QUAN method we got information of the frequency distribution of the various criteria in the ODD diagnosis, and differences between gender and age groups. Through the QUAL method, we got mothers' stories of the most difficult problems they experienced with their children. By mixing the QUAL and QUAN methods and exploring the associations between the two datasets we got a more vivid picture of the children´s behavior behind the criteria of the ODD diagnosis. Thus, it was possible to trace different expressions of externalizing behavior even in children as young as 3–8 years. The difficulties with mixed methods studies are often to find a way to integrate the findings of QUAL and QUAN [39]. The mixed methods (convergent parallel) design used in the present study is similar to the method of collecting information and doing clinical assessment at a children’s psychiatric clinic. Top-down information is gathered when children and parents are asked to fill in various standardized questionnaires, often based on diagnostic criteria. This kind of information is thought to be valid and reliable. The bottom-up approach also gives important information when clinicians ask questions that are more open-ended about the child´s history and context, which are not captured in structured questionnaires. In clinical settings assessments and treatment decisions usually are based on combining both sources of information.

### Limitations

This study had several limitations. Present study has a cross-sectional design and highlights convergences and divergences between top-down and bottom-up approaches concerning symptoms of ODD diagnosis. To really know if the children who exhibited more severe conduct problem in this study later develop conduct disorder (CD), a longitudinal study would be needed. Another important limitation was the small sample, regarding comparisons between boys and girls. In the comparison of themes and categories, our calculation of statistical significance using qualitative data might be considered problematic. However, although the qualitative questions were open-ended, they structured to limit the frames of interpretation. Another limitation was the use of K-SADS as measure for children younger than 6 years (35% of the sample). The diagnostic questions in K-SADS are designed for children aged 6 to 17 years, but the probes and scoring criteria were adapted for children 3–5 years as necessary. This adaptation relied on the three psychologists' experiences in developmental psychology. Participants in the study were mothers only; multiple participants for each child would have provided a fuller description of the children’s functioning. However, by solely interviewing mothers, we got a more homogeneous group. Even if interesting, it was beyond the scope of present study to examine effects of comorbidity on mothers´ description of the children. Furthermore, in an analysis of the most common form of comorbidity in our sample (ODD + ADHD) it did not seem to influence the bottom-up descriptions of the children.

A strength of the study was the interviewer-based diagnostics, in which the clinician decides whether a symptom is present or absent. Many studies use rating scales instead, and respondents (especially parents) usually have limited exposure to the full range of normative behaviors. Another strength was the problem-formulated scales, in which mothers described the most troublesome problems they had with their children, giving an entirely different weight to the problems described. A further strength was the vivid picture of the different behaviors and personality traits hidden behind the diagnostic criteria that were revealed in the qualitative content analysis.

## Conclusions

In the top-down approach, the ODD criteria helped to identify and separate commonly occurring oppositional behavior from conduct problems, but in the bottom-up approach, the accepted diagnostic criteria did not capture the entire range of problematic behaviors-especially those behaviors that constitute a risk for antisocial behavior. The present study shows a gap between the diagnoses of ODD and conduct disorder (CD) in younger children. Antisocial behaviors manifest in preschool and early school years are not always sufficiently alarming to meet the diagnosis of CD, nor are they caught in their entirety by the ODD diagnostic tool. One way to verify suspicion of early antisocial behavior in preschool children would be to specify in the ODD diagnosis if there is also subclinical CD.

## Data Availability

The datasets used and analyzed during the current study are available from the corresponding author on reasonable request.

## Abbreviations

ODD: Oppositional defiant disorder
CD: Conduct disorder
CU-traits: Callous-unemotional traits
LPE: Limited prosocial emotions
DBP: Disruptive behavior problems
QUAL + QUAN: Qualitative + quantitative
